# The evolution, morphology, and development of fern leaves

**DOI:** 10.3389/fpls.2013.00345

**Published:** 2013-09-04

**Authors:** Alejandra Vasco, Robbin C. Moran, Barbara A. Ambrose

**Affiliations:** The New York Botanical GardenBronx, NY, USA

**Keywords:** leaf evolution, megaphyll, plant evo-devo, fronds, pteridophytes, Class I KNOX, Class III HD Zip

## Abstract

Leaves are lateral determinate structures formed in a predictable sequence (phyllotaxy) on the flanks of an indeterminate shoot apical meristem. The origin and evolution of leaves in vascular plants has been widely debated. Being the main conspicuous organ of nearly all vascular plants and often easy to recognize as such, it seems surprising that leaves have had multiple origins. For decades, morphologists, anatomists, paleobotanists, and systematists have contributed data to this debate. More recently, molecular genetic studies have provided insight into leaf evolution and development mainly within angiosperms and, to a lesser extent, lycophytes. There has been recent interest in extending leaf evolutionary developmental studies to other species and lineages, particularly in lycophytes and ferns. Therefore, a review of fern leaf morphology, evolution and development is timely. Here we discuss the theories of leaf evolution in ferns, morphology, and diversity of fern leaves, and experimental results of fern leaf development. We summarize what is known about the molecular genetics of fern leaf development and what future studies might tell us about the evolution of fern leaf development.

## Introduction

“Nature made ferns for pure leaves to show what she could do in that line”

-Henry David Thoreau

**Figure 1:**
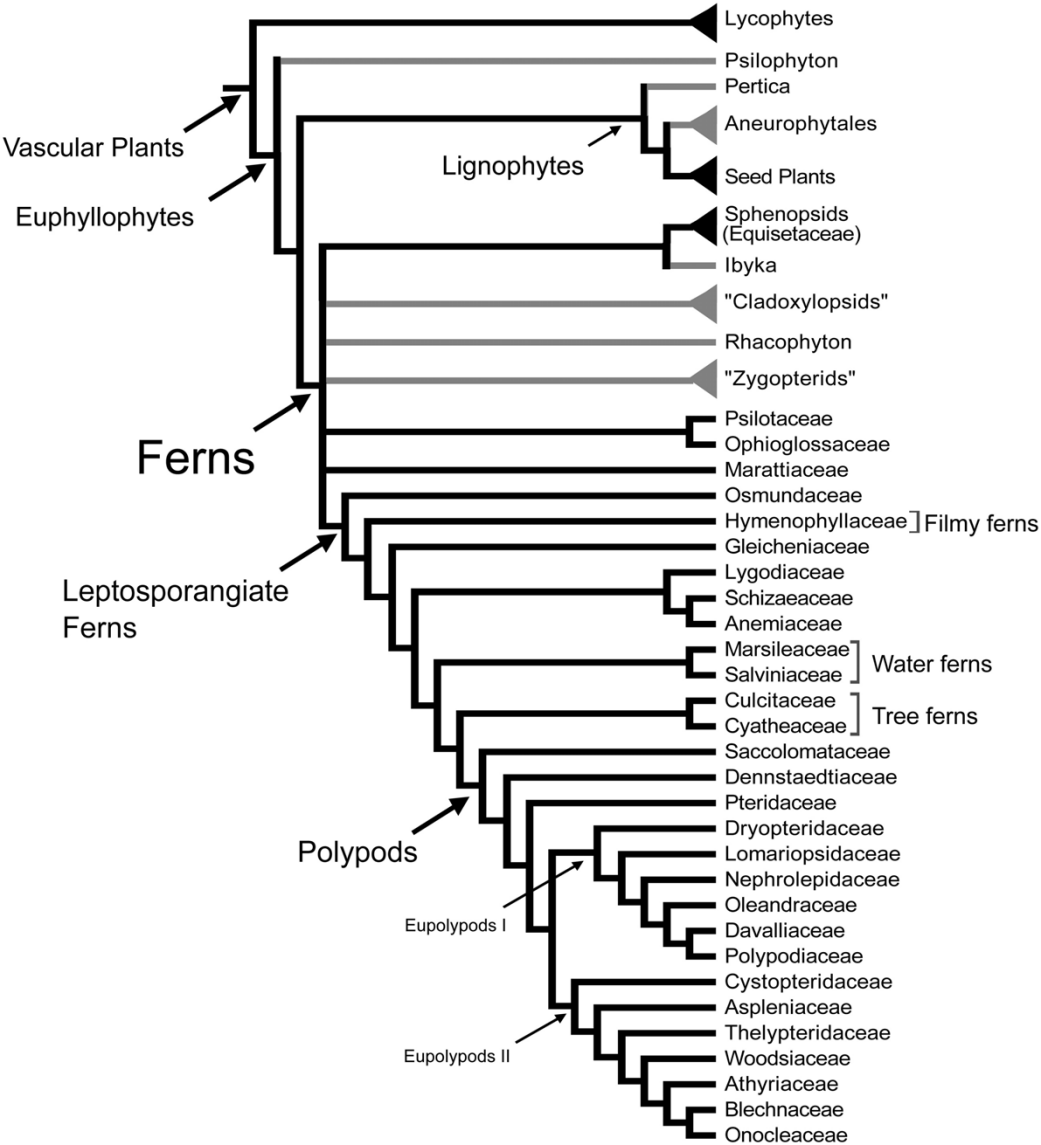
**Relationships between vascular plants with an emphasis on the ferns as summarized from morphological and molecular phylogenetic analyses**. Topology summarizes the results of previously published studies (Kenrick and Crane, 1997; Doyle, 1998; Pryer et al., 2001; Schuettpelz et al., 2007). Branches in gray correspond to exclusively fossil lineages. Names in quotation marks indicate presumed paraphyletic lineages. Only the fern families mentioned in the text are included here, for a complete fern family phylogeny see Smith et al. (2006).

Ferns are the most diverse group of vascular plants after seed plants. Recent morphological and molecular phylogenetic analyses indicate that ferns are the sister group of seed plants (Raubeson and Jansen, 1992; Stevenson and Loconte, 1996; Kenrick and Crane, 1997; Pryer et al., 2001), and include the families Psilotaceae, and Equisetaceae, which have not always been considered as ferns (Figure 1; Pryer et al., 2001). This phylogenetic circumscription and position of ferns has not been accepted by all, especially paleobotanists who have argued that including fossil taxa in the phylogenies resolves ferns as paraphyletic (e.g., Rothwell and Nixon, 2006; Tomescu, 2011). These controversies along with the phylogenetic position of ferns as sister to seed plants, and the fact that fern leaves display a great morphological diversity, make ferns a key plant lineage for comparative studies on how leaves and vascular plants evolved. Studies in ferns are important for resolving morphological interpretations within particular fern groups and are crucial to our understanding of leaf evolution and development in vascular plants.

**Figure 2:**
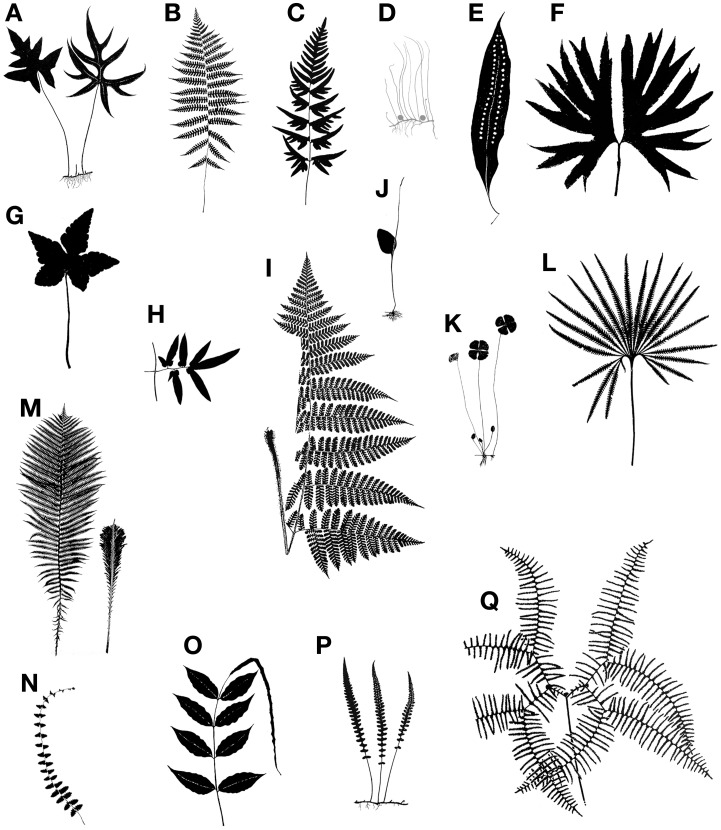
**Examples of the diversity of size and shape in fern leaves. (A)** *Doryopteris nobilis*, pedate laminae. **(B)** *Deparia acrostichoides*, lamina 1-pinnate-pinnatifid. **(C)** *Pteris semipinnata*, dimidiate pinnae.**(D)** *Pilularia globulifera*, filiform, terete leaves attached to rhizome; globular structures are sporocarps. **(E)** *Microgramma megalophylla*, simple and entire lamina. **(F)** *Dipteris conjugata*, lamina divided into two halves from top of petiole. **(G)** *Hemionitis palmata, *palmate lamina. **(H)** *Lygodium flexuosum*, rachis (at right) with lamina of pinnule (other half of pinna not shown). **(I)** *Megalastrum subincisum* (right side of the leaf partially cuted off). **(J)** *Ophioglossum vulgatum*, ovate blade represents a phyllode (expanded rachis). **(K)** *Marsilea drummondii*, lamina consists of two pairs of opposite pinnae, these resembling a four-leaved clover. **(L)** *Matonia pectinata*, lamina. **(M)** *Matteuccia struthiopteris, *sterile-fertile leaf dimorphism (fertile leaf at right). **(N)** *Thelypteris reptans, *flagellate apex proliferous at tip. **(O)** *Bolbitis heteroclita, *1-pinnate lamina with elongate apical segment proliferous at tip. **(P)** *Diplazium tomitaroanum*, pinnatifid leaf. **(Q)** *Gleichenia microphylla*, pair of opposite pinnae.

**Figure 3:**
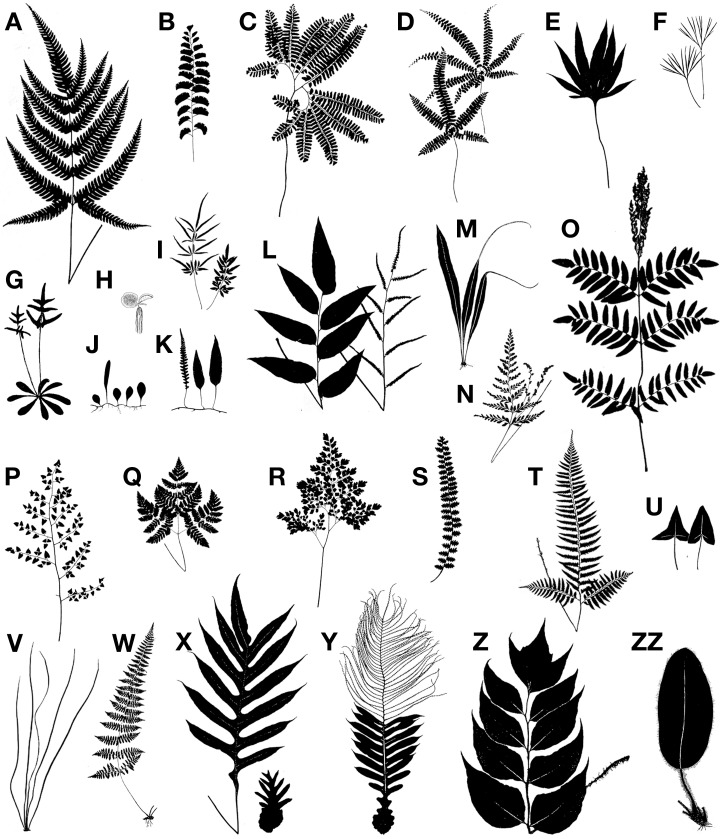
**Examples of the diversity of size and shape in fern leaves. (A)** *Pteris aspercaulis*, enlarged basal pinnules on basiscopic side of basal pinnae. **(B)** *Adiantum lunatum*, 1-pinnate. **(C)** *Adiantum pedatum, *pedate. **(D)** *Adiantopsis radiata*, digitate pinnae. **(E)** *Pyrrosia polydactyla, *palmately lobed. **(F)** *Actiniopteris semiflabellata*, incised leaf. **(G)** *Trachypteris pinnata*, holodimorphic, with rosette of sterile leaves and erect fertile ones. **(H)** *Salvinia molesta, *root-like (lower) leaf is submerged and bears sori, the two round ones are floating. **(I)** *Pteris ensiformis, *holodimorphic, fertile leaf at left. **(J)** *Lemmaphyllum microphyllum*, holodimorphic, longer leaf is fertile. **(K)** *Davallia heterophylla, *holodimorphic, fertile leaf at left. **(L)** *Olfersia cervina, *holodimorphic, fertile leaf at right. **(M)** *Belvisia mucronata, *hemidimorphic, with caudate fertile apex. **(N)** *Anemia adiantifolia, *hemidimorphic, with two basal pinnae fertile and long-stalked. **(O)** *Osmunda regalis, *hemidimorphic, with fertile pinnae at apex. **(P)** *Pellaea cordifolia, *decompound. **(Q)** *Gymnocarpium robertianum, *ternate. **(R)** *Adiantum raddianum, *decompound. **(S)** *Astrolepis sinuata, *1-pinnate-pinnatifid. **(T)** *Polystichum tripteron, *enlarged basal pinnae. **(U)** *Hemionitis ariifolia*, hastate at left, deltate at right (from same plant). **(V)** *Vittaria lineata, *linear leaves (shoe-string fern). **(W)** *Cystopteris bulbifera, *long-attenuate apex. **(X)** *Drynaria quercifolia, *debris-collecting leaf at right. **(Y)** *Aglaomorpha meyeniana*, hemidimorphic, with narrow distal pinnae fertile and base expanded for collecting fallen organic debris. **(Z)** *Cyrtomium macrophyllum, *1-pinnate. **(ZZ)** *Elaphoglossum crinitum, *simple, entire.

Leaves have been the center of many evolutionary and developmental studies, because they are the dominant, most conspicuous organs of most plants, including ferns. Although typically envisioned as compound, the leaves of ferns actually display great morphological diversity (Figures 2, 3). Historically, the leaves of the eusporangiate Marattiaceae and of leptosporangiate ferns (Figure 1) have been considered megaphylls, but there has been great controversy on the definition of leaves in the Psilotaceae, Ophioglossaceae, and Equisetaceae (e.g., Chrysler, 1910; Sen, 1968; Bierhorst, 1971; Kato, 1988; Kenrick and Crane, 1997). These families have extremely modified leaves, and this has made their interpretation difficult. If ferns are considered a monophyletic group (Figure 1), then all fern leaves are considered to be megaphylls or at least derived from megaphyllous ancestors. Megaphylls then are present in seed plants and ferns and there are several competing theories regarding their evolution and origin. It is still an open question whether the leaves of all fern are homologous, let alone whether fern leaves are homologous with seed plant leaves.

Here we review the following about fern leaves: their evolution, their general morphology and diversity, unusual adaptations, the experimental biology, and the molecular genetic studies to date. Integrating these different fields will not only shed light on fern leaf evolution and development and help refine hypotheses of fern leaf evolution, but also further our understanding of leaf evolution and development across the vascular plants.

## The evolution of leaves in ferns

In general, leaves are the main conspicuous organs of vascular plants and often easy to recognize as such. It may therefore seem surprising that they have had multiple origins. It has been hypothesized that leaves evolved once in the ancestor of all vascular plants (Kaplan, 2001; Schneider et al., 2002), twice (microphylls in lycophytes and megaphylls in the remaining vascular plants; Bower, 1935), three times (separately in lycophytes, ferns, and seed plants; Kenrick and Crane, 1997; Friedman et al., 2004; Galtier, 2010; Corvez et al., 2012), four times (Boyce and Knoll, 2002), or six or more times (Tomescu, 2009). The number of times leaves are thought to have evolved separately in vascular plants depends on the phylogenetic hypothesis used and the inclusion and morphological interpretations of fossil taxa (Boyce, 2010).

Currently there is a general consensus that the leaves of lycophytes and euphyllophytes are not homologous and have evolved independently (Kenrick and Crane, 1997). Most morphological and molecular phylogenetic analyses including either living or fossil taxa or both, indicate that lycophytes are the sister group of all other extant vascular plants, which are also called euphyllophytes (Raubeson and Jansen, 1992; Stevenson and Loconte, 1996; Kenrick and Crane, 1997; Pryer et al., 2001; Schneider et al., 2009) (Figure 1). Several studies have pointed out that the earliest fossil relatives of both lycophytes and euphyllophytes were leafless. If this is the case, then these two groups of plants must have evolved leaves independently (Kenrick and Crane, 1997; Friedman et al., 2004; Boyce, 2010).

**Figure 4:**
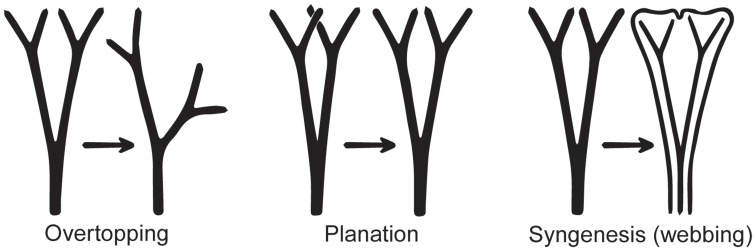
**Three processes from the telome theory proposed to be involved in megaphyll evolution (redrawn from Zimmermann, 1952)**. These processes may have occurred in various sequences in different groups of vascular plants.

The leaves of euphyllophytes have been called megaphylls (Gifford and Foster, 1988), and there are several competing theories regarding their evolution. The most widely accepted is the one proposed by Zimmermann (1930). Concerning the evolution of leaves, his telome theory proposed that megaphyll evolution involves three elementary processes that could occur independently of each other (Figure 4). These three processes transformed the dichotomous, leafless, photosynthetic axes of the sporophyte of early Devonian plants into leaves (Zimmermann, 1930, 1952; Wilson, 1953; Stewart, 1964). One process involved the elongation of some of the branches more than others, producing a main central branch with subordinate lateral ones (overtopping). A second process involved flattening the branching system into one plane (planation). A third process involved the development of laminar tissue between the axes (syngenesis or webbing). Paleobotanical evidence supports the existence of these different processes and also suggests that there were anatomical changes that correlated with the origin of megaphylls (Galtier, 2010). A fourth process, reduction of the branch system to single scale-like leaf, was proposed to explain how leaves in *Psilotum* (Psilotaceae) and lycophytes evolved (Zimmermann, 1930, 1952; Wilson, 1953).

Determining homology of megaphylls among euphyllophytes is challenging because the three processes of megaphyll evolution—overtopping, planation, and webbing—could have developed independently at different times and in different orders. Hypotheses of megaphyll homology depend on the morphological and anatomical interpretations of extinct leafless Devonian and Carboniferous plants. One hypothesis suggests that megaphylls are not homologous within the euphyllophytes. This is based on the phylogenetic placement of leafless fossils, such as *Psilophyton* and *Pertica*, within the euphyllophytes, and the placement of the Aneurophytales among lignophytes (Figure 1). Such topology indicates that the common ancestor of lignophytes (seed plants+fossil Aneurophytales) and that of ferns were leafless (Kenrick and Crane, 1997; Friedman et al., 2004; Boyce, 2010; Corvez et al., 2012). Another hypothesis proposes that megaphylls in the euphyllophytes may be homologous at the level of lateral branches (megaphyll precursors), and that the processes of flattening into one plane (planation, which sometimes also has been suggested to imply abaxial/adaxial anatomical organization of leaves) and the formation of a lamina (webbing) developed independently in ferns and seed plants (Kenrick and Crane, 1997; Galtier, 2010). This hypothesis is based on the statement that the earliest known megaphyll-like structures are highly dissected and composed of segments that were short, narrow, and single-veined, but lacked an expanded lamina and the abaxial/adaxial anatomical organization of leaves (Rothwell, 1999; Boyce and Knoll, 2002). Fossil evidence has also allowed some authors to hypothesize that the full set of megaphyllous traits were acquired later, either at the same (Boyce and Knoll, 2002) or in a different (Sanders et al., 2009) sequence of events in ferns compared to seed plants.

Even within ferns the homology of leaves is unclear. There is currently no consensus whether the leaves of major fern clades such as Equisetaceae (horsetails), Psilotaceae, Ophioglossaceae, Marattiaceae, and leptosporangiate ferns are homologous (Figure 1). There are three main causes for this uncertainty. One is conflicting phylogenetic hypotheses. Molecular phylogenetic hypotheses of extant taxa consider ferns as a monophyletic group that includes the Equisetaceae, Psilotaceae, and Ophioglossaceae (Figure 1; Pryer et al., 2001; Qiu et al., 2006; Grewe et al., 2013). In contrast, morphological phylogenetic hypotheses that incorporate fossils and extant taxa place Equisetaceae, Psilotaceae, and Ophioglossaceae in different positions within the vascular plants, related to but not as part of the ferns (Rothwell, 1996, 1999; Stevenson and Loconte, 1996; Rothwell and Nixon, 2006). If the molecular phylogenetic hypothesis is accepted, then all ferns leaves should be homologous at some level. In contrast, if the morphological phylogenetic hypotheses are accepted, then the leaves of ferns, Equisetaceae, Psilotaceae, and Ophioglossaceae are not necessarily homologous. The second reason for the uncertainty about megaphyll homology in ferns is that there are conflicts about the interpretation and codification of characters of extinct Devonian and Carboniferous fernlike plants without laminate leaves. These conflicts make the phylogenetic placement of the fossils equivocal, and therefore statements of leaf homology within ferns ambiguous. For instance, although they did not analyze ferns in detail, Kenrick and Crane (1997) considered modern ferns, sphenopsids, cladoxylopsids, and the Devonian genus *Rhacophyton* as part of the same clade (Figure 1). However, Rothwell (1996, 1999) separated living ferns from *Rhacophyton* and zygopterids, which he placed with lignophytes based on secondary growth. This topology (not shown in Figure 1) would mean that no Devonian members of the line leading to modern ferns and no steps in the origin of the fern leaf are known. The third reason for the difficulties is that no fossils have been associated with ancestors of key lineages within ferns such as Ophioglossales and Psilotales, whose extremely modified leaves could suggest that they are not homologous to those of other ferns. The lack of these fossils means that there is no evidence concerning the mode of origin of their leaves. For the Marattiales, which has an extensive fossil record from the Carboniferous, all the fossils have planated leaves, thus giving no evidence as to the sequence of steps in how their leaves evolved (Rothwell and Stockey, 1989; Rothwell, 1996). Fossil evidence supports the view that *Equisetum* (sphenopsids) leaves were derived from a single dichotomous branch (Lignier, 1908 in Kenrick and Crane, 1997), and that Marattiales and leptosporangiate ferns have basically compound leaves more probably derived from whole branch systems bearing dichotomous appendages (Doyle, 2013).

Currently the fossil record is insufficient to infer what intermediate characteristics of megaphylls may be homologous between different fern lineages. Insights about this topic will probably come from studies of morphology, developmental pathways, and genetic networks of living taxa with different leaf morphologies within a phylogenetic context (e.g., Stevenson and Loconte, 1996; Harrison et al., 2005; Rothwell and Nixon, 2006). Even though there is no complete sequenced genome or functional model system for ferns, there is now a considerable amount of transcriptome data for ferns available through the 1 KP project (http://www.onekp.com/). This evidence, along with studies designed to understand the molecular genetic basis of fern leaf diversity will provide crucial data on leaf developmental pathways in ferns and allow us to refine existing hypotheses on fern leaf evolution.

## The general morphology of fern leaves

Although primarily for photosynthesis, fern leaves may also assume other tasks such as propagating the plant vegetatively by bulblets, harboring nitrogen-fixing cyanobacteria, forming nests that collect humus falling from above, or efficiently dispersing spores. Furthermore, ferns grow in many habitats—from mangroves at sea level to alpine vegetation above tree line, temperate forests to arctic tundras, and desserts to wetlands. Given this great diversity in functions and habitats it is not surprising that fern leaves exhibit a great diversity in size and shape (Figures 2, 3). This morphological diversity has helped taxonomists and morphologists understand the evolution of ferns, but it can also be used as a tool to unravel the developmental pathways underlying fern leaf evolution.

**Figure 5:**
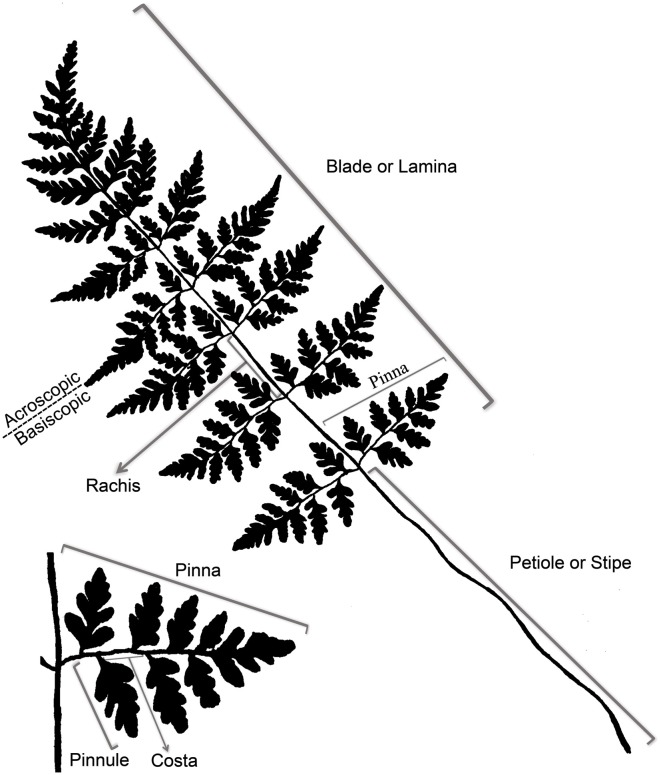
**Terminology of a typical fern leaf or frond**.

In nearly all extant ferns, leaves constitute the dominant organs of the plant. Fern leaves are typically envisioned as compound (also termed dissected or divided) with pinnae or pinnules arranged along a central axis (the rachis or costa) (Figure 5). Most people probably envision ferns this way because, in fact, most fern leaves *are* highly divided. Yet fern leaves exhibit enormous diversity, especially in size, shape, and cutting (Figures 2, 3).

Even though there is a great diversity of fern leaves, all share common characteristics and consist basically of a stalk and a lamina (see unusual fern leaves section for exceptions). The terms applied to parts of a typical fern leaf differ sometimes from the ones applied to seed plant leaves; thus, it is helpful to review the terminology of a typical fern leaf (Figure 5). Fern leaves are often called fronds, the stalk of the leaf is called the stipe or petiole. Distal to this, the laterally expanded portion of the leaf is termed the blade or lamina, whose central midrib is referred to as the rachis. The primary divisions of the blade are called pinnae, and the secondary segments of these (if present) are called pinnules. The stalk of a pinna could be called a petiolule, but this term is seldom used in fern taxonomy. If the pinnules are further divided, the divisions are termed segments, and sometimes their order is specified, such as “tertiary segment” or in a more highly divided leaf, “quaternary segment.” The midrib of the pinna is termed a costa, and the midrib of a pinnule is called the costule. That half of a pinna or pinnule that occurs on the side toward the distal apex of the axis that bears it is called the acroscopic side, and accordingly, the side that occurs toward the base is the basiscopic (for more details of fern leaf terminology, see Tryon, 1960).

Fern leaves and megaphylls of other groups are defined by a combination of characters that are a result of specific developmental processes. They are arranged in a fixed and predictive phyllotaxy around the shoot apex (Schoute, 1938; Gifford and Foster, 1988), they have adaxial/abaxial identities and with laminar tissue supplied by numerous vascular traces, and most grow for a finite amount of time to a finite size (Bower, 1923; Hagemann, 1989; Kaplan and Groff, 1995; Kaplan, 2001).

Most seed plants (cycads excepted; Stevenson, 1990) have buds that form in the axils of the leaves as they are specified from the shoot apical meristem and can subsequently grow out as shoots (branches). However, nearly all ferns have extra-axillary branching, meaning that buds that will grow out as shoots may take various positional relationships with respect to the point of leaf insertion on the stem (Hagemann, 1989). Axillary branching is extremely rare in ferns and, where investigated anatomically, the vasculature of the leaves differs from that found in seed plants because the steles do not come directly from the axil or are closely associated with the leaf gap (Hébant-Mauri, 1984; Hagemann, 1989).

Nearly all mature fern leaves are bifacial, with well-defined adaxial/abaxial identities. The condition is also present anatomically two ways: first, with the mesophyll differentiated adaxially as palisade tissue and abaxially as spongy tissue, and second, by greater elongation of cells on the abaxial side vs. adaxial. A few ferns, such as *Enterosora* (Polypodiaceae) have a spongy mesophyll throughout, but this is rare among ferns (Moran, pers. obs.). One exception to a mature bifacial leaf is *Pilularia* (Marsileaceae, water ferns), whose leaves are terete and unifacial. Its blade-less leaf is interpreted as a petiole that has lost its apical pinnae (Eames, 1936). At least in fossil ferns, the defined adaxial/abaxial identities are also present in the vascular bundles of the petiole where the protoxylem is adaxial (in contrast to seed plants that have abaxial protoxylem; Galtier, 2010).

**Figure 6:**
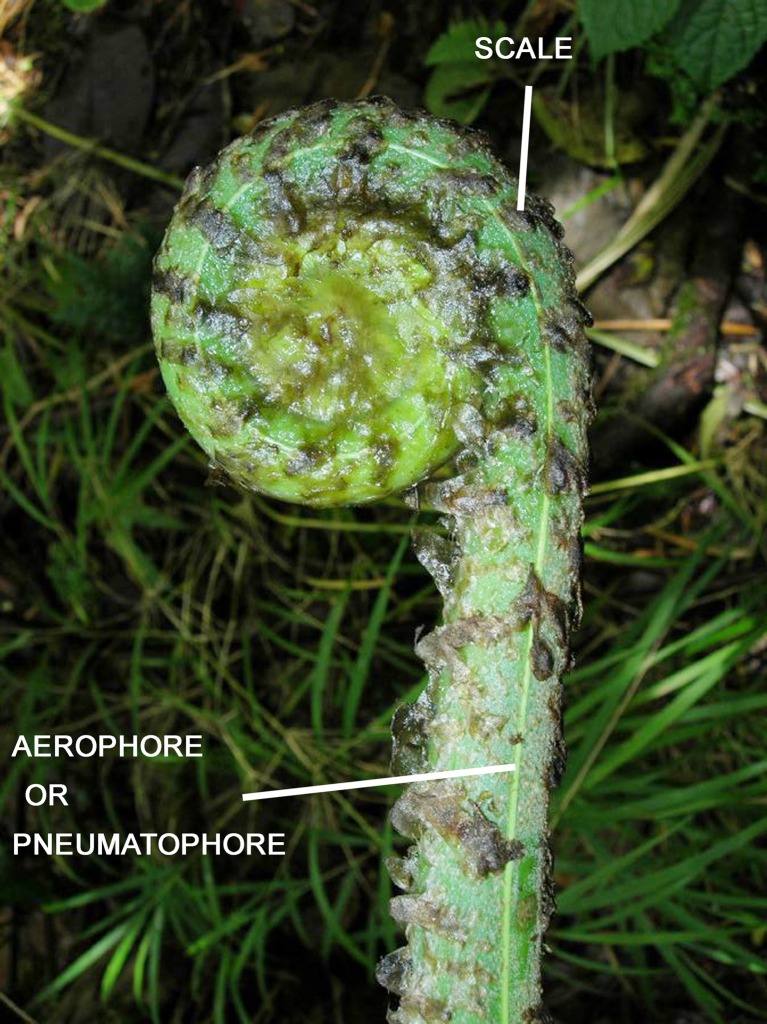
**Fiddlehead and aerophore (or pneumatophore) of *Pteris livida* (Pteridaceae)**.

Besides the typical characteristics of all megaphylls, fern leaves have some additional characteristics that make them distinctive. Their leaf primordia are often covered by hairs and/or scales (Figure 6). Hairs are uniseriate and either one-celled or multicelled. Hairs develop from cell divisions of a single epidermal cell (Bower, 1923). In contrast, scales are multicellular with the cells arranged side-by-side in two or more rows. They develop from cell divisions of several epidermal cells. Scales can be persistent in mature leaves and may become smaller and reduced to uniseriate proscales toward the margins of the laminae (Moran, 1986). Such highly reduced, uniseriate scales are sometimes also called microscales (Daigobo, 1972).

Another characteristic of fern leaves is that all cells of the lower and upper epidermis contain chloroplasts (Copeland, 1907; Wylie, 1948a,b, 1949). This condition is shared with the lycophytes (Moran, pers. obs.). In contrast, seed plants (with few exceptions) have chloroplasts only in the guard cells of the epidermis, not in other epidermal cells (Cutter, 1978; Gifford and Foster, 1988; Fahn, 1990). Among ferns, the few exceptions that lack chloroplasts in all epidermal cells are species that grow in full sun, such as high-canopy epiphytes (e.g., *Elaphoglossum lingua, *Dryopteridaceae) or on sunny rock faces (e.g., *Notholaena affinis, *Pteridaceae; Moran, pers. obs.). The absence of chloroplasts from the epidermis of seed plants (except the guard cells) is best interpreted on the basis of outgroup comparison as a loss, and this loss represents a synapomorphy for seed plants.

There are two characteristics typical of most fern leaves: a fiddlehead and aerophore lines (Figure 6). Fiddleheads, also called croziers are circinately coiled leaf buds. Just as the whole leaf is coiled in bud, so too are its subdivisions, the pinnae and pinnules. Presumably, the function of coiling is to protect the soft meristematic parts concealed within the fiddlehead. Fiddleheads are highly distinctive of ferns because they are absent from lycophytes and nearly all seed plants (some exceptions in seed plants are the cycads *Cycas* and *Stangeria, *and the insectivorous plant *Drosophyllum lusitanicum, *although in the latter the coiling is abaxial, not adaxial as in ferns). However, some ferns such as *Psilotum, Equisetum*, *Ophioglossum*, *Salvinia*, and *Azolla*, lack fiddleheads. Aerophores or pneumatophores are also characteristic of nearly all leptosporangiate fern leaves. They are apparent as two light-colored lines on either side of the petiole (Davies, 1991). These lines aerate the leaf. Their surfaces bear stomata that allow air to diffuse into the loosely packed parenchyma beneath the line. In some ferns the aerophores extend distally into the rachis, or proximally onto the rhizomes (e.g., *Mickelia* and *Polybotrya*, both Dryopteridaceae). In those few fern species that invest their fiddleheads in a thick covering of mucilage, the lines are modified at the pinna bases into elongate peg-like structures that protrude through the mucilage, thus aerating the developing leaf (Hennipman, 1968). Even when aerophore lines are seemingly absent, such as in the darkly sclerotized petioles of *Adiantum pedatum* (Pteridaceae, Figure 3C), a line of stomata are present in its place—a vestige of the ancestral aerophore.

## The morphological diversity of fern leaves

There is a vast amount of leaf diversity in ferns, and here we give only a few examples (Figures 2, 3). Fern leaf morphological diversity is exhibited in the lamina size, shape, dissection, reductions in various parts, apical indeterminacy, petiole vasculature, and petiole morphology.

### Lamina dissection and shape

**Figure 7:**
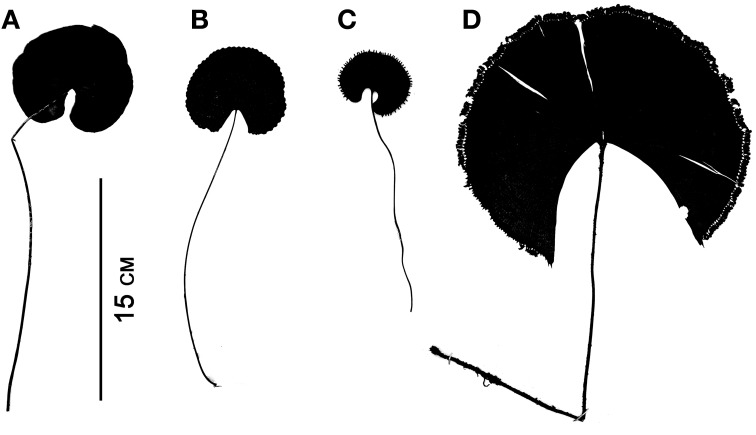
**Parallelism in four simple-leaved ferns belonging to different families. (A)** *Lindsaea cyclophylla* (Lindsaeaceae). **(B)** *Adiantum reniforme* (Pteridaceae). **(C)** *Trichomanes reniforme* (Hymenophyllaceae). **(D)** *Schizaea elegans* (Schizaeaceae).

Although fern leaves are often stereotyped as being finely divided, some are simple and entire, and others are merely lobed. Simple leaves in ferns are found in *Elaphoglossum* (ca. 600 spp.; Dryopteridaceae), *Campyloneurum* (ca. 50 spp.; Polypodiaceae), *Microgramma* (35 spp.; Polypodiaceae), *Grammitis* (22 spp.; Polypodiaceae), and *Vittaria* (6 spp.; Pteridaceae). On the basis of outgroup comparison, the leaves of these ferns are believed to be derived from ancestors with more divided leaves (e.g., Wagner, 1964; Moran et al., 2010, for *Elaphoglossum*). One example of simple leaves are the reniform ones that have evolved independently in different fern families, resulting in some striking parallelisms (Figure 7). This ability to produce similarly shaped laminae suggests similar developmental mechanisms may be at work. Lobed leaves are also found in ferns, and the basic plan may be either pinnate, as in *Aglaomorpha meyeniana* (Polypodiaceae, Figure 3Y), or palmately lobed as in *Hemionitis palmata* (Pteridaceae, Figure 2G). Not surprisingly divided fern leaves exhibit a great variety of architectures. The most common and widespread is the pinnate plan. Here the petiole continues into the lamina as a single, unbranched rachis that produces lateral pinnae. Examples are *Deparia acrostichodes* (Thelypteridaceae, Figure 2B), *Matteuccia struthiopteris* (Onocleaceae, Figure 2M), and *Megalastrum subincisum* (Dryopteridaceae, Figure 2I). Sometimes the basal pinnae are repeatedly branched on the basiscopic side—a condition known as pedate. Examples are *Adiantum pedatum* (Pteridaceae, Figure 3C) and *Doryopteris nobilis* (Pteridaceae, Figure 2A). In highly divided leaves, branching patterns of the pinnae (primary divisions of the laminae) may be of taxonomic importance (e.g., species of *Megalastrum* (Figure 2I) and *Lastreopsis, *both Dryopteridaceae).

### Leaf indeterminacy

**Figure 8:**
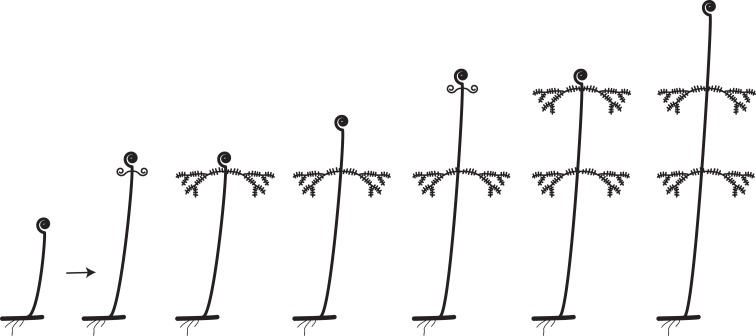
**Rhythmic leaf extension growth in the Gleicheniaceae (from Moran, 2004)**.

Leaves are generally considered to be determinate organs; that is, they grow only to a certain length and no more. Fern leaves generally exhibit finite (determinate) growth but with longer meristematic activity of the apical portion and maturation toward the apex (acroscopic growth). This prolonged meristematic activity is due to an apical cell at the leaf tip and is a distinctive feature of ferns compared to seed plants (Imaichi, 2008). A few ferns, however, have indeterminate leaves. Generally such leaves are either pendulous or scrambling over the surrounding vegetation. *Alansmia* (Polypodiaceae) is a pendent epiphyte whose leaves exhibit small continuously growing fiddleheads at their tips (Kessler et al., 2011). Their laminae often exhibit constrictions where fiddlehead activity diminishes during a less favorable part of the growing season. Many species of *Nephrolepis* (Nephrolepidaceae), particularly *N. exaltata* (Boston fern) and *N. pendula, *exhibit pendulous indeterminate leaves. The latter species can have leaves up to three meters long (Hovenkamp and Miyamoto, 2005; Rojas-Alvarado, 2008). Ferns with scrambling indeterminate leaves grow over the surrounding vegetation and use it for support. Examples include certain species of Gleicheniaceae (Figures 2Q, 8; Moran, 2004), *Jamesonia* (Pteridaceae, Tryon, 1970), and *Hypolepis* (Dennstaedtiaceae, Brownsey, 1987; Holtum, 1958). Scrambling ferns often have leaf apices exhibiting intermittent growth (Figure 8). The leaf apex (fiddlehead) rests while the subtending lateral pinnae develop. After extending laterally, the pinnae come to rest on the surrounding vegetation, and the leaf apex resumes extension growth. In this manner, and because of their long-creeping rhizomes, the Gleicheniaceae often form dense extensive thickets (Moran, 2004). Two genera of ferns that have indeterminate leaves twining around a support are *Salpichlaena* (Blechnaceae) and *Lygodium* (Lygodiaceae, Figure 2H; Mueller, 1983a,b). In Costa Rica, the leaves of *Salpichlaena volubilis* are generally 10–12 m long, whereas those of *Lygodium venustum* are usually 3–6 (Moran, pers. obs.). In these genera it is the rachis that twines—a condition not found among the angiosperms (the organ that twines in angiosperms such as *Wisteria* (Fabaceae) or *Convolvulus* (Convolvulaceae) is the stem). The twining results from the widely circumnutating leaf being interrupted by contact with a support, and, when the rope hits a pole, the rope continues its motion around the pole, wrapping upward and thus climbing the pole (Darwin, 1876). This is not a thigmotropic grasping response as exhibited, for instance, by the leaves of *Clematis* (Ranunculaceae; Darwin, 1876).

### Petiole vasculature

**Figure 9:**
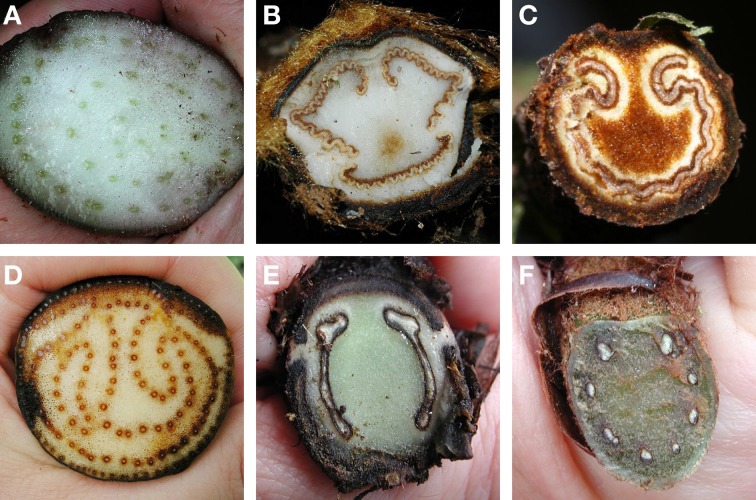
**Examples of petiole vasculature in ferns, as seen in cross section. (A)** *Eupodium laeve* (Marattiaceae). **(B)** *Dicksonia sellowiana* (Dicksoniaceae). **(C)** *Saccoloma chartaceum* (Saccolomataceae). **(D)** *Acrostichum danaeifolium* (Pteridaceae). **(E)** *Diplazium hians* (Eupolypods II, Athyriaceae). **(F)** *Polystichum concinnum* (Eupolypods I, Dryopteridaceae).

Ferns exhibit a wide variety of petiole vasculatures (Figure 9). One extreme is the polycyclic condition found in *Acrostichum* (Pteridaceae) and the Marattiaceae. Here the petioles contain several concentric circles (as seen in transverse section), each circle composed of many individual leaf traces (Figure 9A). At the other extreme is, for instance, *Trichomanes* (Hymenophyllaceae, filmy ferns), whose petioles contain only one vascular bundle. A single omega shaped vascular bundle, with the open end of the omega oriented adaxially, is found in Culcitaceae, Dennstaedtiaceae, Saccolomataceae (Figure 9C), and many species of *Pteris* (Pteridaceae). The number and arrangement of vascular bundles in the petiole is helpful in fern taxonomy. Of great importance in fern taxonomy is the distinction between Eupolypods I and II (i.e., about 65% of extant ferns) based on petiole vasculature. With few exceptions, Eupolypods I have several vascular bundles, all circular in cross section, with the two adaxial ones enlarged (Figure 9F), whereas Eupolpods II have only two vascular bundles elongated in cross section (Figure 9E; Moran, pers. obs.).

### Petiole morphology

In some ferns only the very base of the petiole develops and the distal portion of the leaf does not. In these cases the distal part of the leaf is represented by either a vestigial fiddlehead or necrotic tissue. In *Matteuccia struthiopteris* (Onocleaceae, Figure 2M) and various species of *Osmunda* (Osmundaceae), petiole bases are called cataphylls, and they protect the rhizome apex (Goebel, 1905). In other ferns, the leaf bases store abundant starch and are termed trophopods (Wagner and Johnson, 1983). These structures occur in *Cystopteris protrusa* (Cystopteridaceae) and *Onoclea sensibilis* (Onocleaceae). In some species of *Diplazium* (Athyriaceae) with erect subarborescent rhizomes (e.g., *Diplazium prominulum* and *D. striatastrum*), the rigid, starch-filled trophopods are tightly appressed to the trunk and structurally support the rhizome (Moran, pers. obs.).

## Ferns with unusual leaf morphologies

Some fern leaves present unusual shapes and adaptations. The subject is vast and only a small sample is covered here.

### Ophioglossaceae

Relative to other ferns, unusual features of the leaves of this family include sheathing leaf bases, buds rarely circinate, and adaxial position of the sporangia (Bower, 1926; Kato, 1988). Furthermore, species of Ophioglossaceae usually produce only one leaf at a time. The principal attribute distinguishing Ophioglossaceae from other ferns is the division of the leaf into separate vegetative (sterile segment) and sporangium-bearing (fertile segment) portions (Figure 2J; Bower, 1926; Gifford and Foster, 1988; Wagner, 1990). The fertile segment has been variously interpreted. One interpretation is that it develops from a single pinna or two fused pinnae so that the plant is composed of a leaf with a sterile portion that is proximal and a fertile portion that is distal (Chrysler, 1910, 1911). Another interpretation is that the fertile spike is derived from a shoot or a modified branch system so that the plant consists of an organ that is intermediate between a true leaf and a branch (Zimmermann, 1952; Wilson, 1953; Sen, 1968). The sterile segment of *Ophioglossum* (Figure 2J) lacks a midrib, and this has been proposed to be the result of the loss of the lamina tissue and retention of the rachis. The latter expanded laterally by intercalary divisions and, at the same time, elaborated a system of reticulate veins (Wagner, 1990). In other words, the lamina of *Ophioglossum* is a phyllode.

### Psilotum

The sterile leaves of *Psilotum* (Psilotaceae) are unusual among ferns by being scale-like and generally lacking a vascular supply. The “simple” structure of the leaves and the dichotomizing axes that constitute the entire plant, led several authors to relate *Psilotum* to the earliest vascular plants (Bower, 1935; Eames, 1936; Wilson, 1953; Rothwell, 1999). Molecular phylogenetic analyses of chloroplast and nuclear markers (Pryer et al., 2001) and from complete plastid genomes (Grewe et al., 2013), however, have confirmed that Psilotaceae are ferns (Figure 1). The inclusion of Psilotaceae within the ferns suggests that the small scale-like appendages in *Psilotum* are probably best interpreted as highly reduced leaves.

### Equisetum

The leaves of *Equisetum* (Equisetaceae) are unique among ferns in form and function. Unlike most ferns that have leaves as the dominant organs of the plant, in *Equisetum* the stem is the dominant organ and performs most of the plant's photosynthesis. The leaves are highly reduced and connate laterally to form a sheath around the base of each segment of the aerial stem. The teeth along the rim of the sheaths represent free leaf tips (in some species, such as *E. hyemale*, the teeth are deciduous). At the base of each leaf sheath is a node at which there is an intercalary meristem. New stem tissue produced by these meristems is responsible for nearly all of the elongation of the aerial stem. In temperate species, the apical meristem differentiates as parenchyma by the end of the summer and therefore is no longer active. In the following spring, nearly all the elongation of the aerial shoots comes from the activity of the intercalary meristems (Hauke, 1985). At each node, the youngest—and therefore softest and structurally weakest—stem tissue is located just above the intercalary meristem and surrounded by the leaf sheath. The function of the sheath is to structurally support this weaker stem tissue, just as leaf sheaths do in other plants with intercalary meristems at the nodes, such as grasses, sedges, and commelinid monocots (Fisher and French, 1976). In *Equisetum*, the relationship of the vegetative and fertile portions has been debated: the sporangiophore (the fertile portion) has been interpreted as being a novel organ “(organ sui generis)” or homologous to the leaves (Goebel, 1905; Bower, 1935; Zimmermann, 1952; Page, 1972). Complete serial transitions between the leaf sheaths and the sporangium-bearing structures have been found, suggesting that the latter are sporophylls (Page, 1972).

### Filmy ferns

In the filmy ferns (600 spp., Hymenophyllaceae), the laminae are one cell layer thick between the veins. A few species are known with 2–4 cell layers, but these species lack intercellular spaces and stomata (Copeland, 1938). False veins occur in some filmy fern genera, such as *Didymoglossum* (Wessels Boer, 1962). These veins are one cell wide and appear as faint streaks that do not connect to the true veins. The thin laminae of filmy ferns dry out readily and then, upon rehydration, rapidly expand and resume photosynthesis (Proctor, 2003, 2012). They are capable of directly absorbing water and nutrients.

### Heterosporous water ferns

The leaves of all heterosporous water ferns are unusual, having been highly modified for their aquatic or semi-aquatic habitats. *Salvinia* (Salviniaceae) has two kinds of leaves produced in a false whorl of three (Croxdale, 1978, 1979, 1981). The first kind of leaf, of which there are two in the false whorl, is floating, green, entire, and conduplicate in bud (i.e., not circinate). The other kind of leaf is whitish, highly divided, and hanging down in the water. Only this submerged leaf bears sori. The dorsal surfaces of the floating leaves are covered by erect papillae. In some species (e.g., *S. auriculata* and *S. molesta, *Figure 3H) these papillae have four hairs at their apices, the complete structure (papillae and hairs) resembling an egg-beater. These peculiar structures help water bead-up and roll off the leaf. The surface of the leaf, except for those apical cells of hairs united by their tips, bear a super-hydrophobic wax that prevents wetting the surface, thus helping to keep the plant afloat (Barthlott et al., 2010). *Azolla* has bilobed leaves up to 1 mm long—the smallest leaves of any fern. A colorless ventral lobe rests on the water and a thicker green dorsal lobe arches upward. This thicker lobe contains a cavity that harbors nitrogen-fixing cyanobacteria (*Anabaena azollae*). *Marsilea* (Marsileaceae, Figure 2K) is unusual because its leaves resemble a four-leaved clover. Its leaflets (pinnae) consist of two pairs of opposite pinnae, each pinna provided with a pulvinus at its base. Through the action of the pulvini, the pinnae fold forward and upward at night, forming a vertical packet with the basal pair enclosed within the distal one. At dawn the packet unfolds to present the pinna perpendicularly to the sun (Darwin, 1896). *Marsilea* is the only nyctinastic fern. Also unusual about *Marsilea* (and the other two genera of Marsileaceae, *Pilularia, *and *Regnellidium*) are its sporocarps, hardened bean-like structures that represent folded and marginally sealed pinnae containing the sori (Eames, 1936; Nagalingum et al., 2006).

### Tree ferns

Among the tree-fern family Cyatheaceae, several species of *Alsophila* produce at the base of their petioles skeletonized pinnae with linear or filiform segments. They bear stomata on their abaxial surfaces and have compact mesophyll with little intercellular spaces (Goebel, 1905). These pinnae, called aphlebiae, are of unknown function.

### Drynarioid ferns

As an adaptation to their epiphytic life style, the drynarioid genera of Polypodiaceae have leaves modified for collecting organic debris that falls from above, mostly bits of bark and leaves (Hennipman and Roos, 1982; Janssen and Schneider, 2005). As the debris decomposes, it forms humus into which the plant grows roots to absorb water and nutrients. This is important nutritionally because these ferns are epiphytic, not in contact with water and minerals in the soil. One of these genera of humus collectors, *Aglaomorpha* (Polypodiaceae, Figure 3Y), has sterile and fertile leaves of one type (monomorphic) but modified within the same leaf to accumulate organic debris. The leaves are sessile with deeply pinnatifid laminae and expanded bases that turn brown and papery with age. The bases resist decay and persist on the plant long after the green laminar tissue has decayed away (the rachises are persistent, though). Thus, the old leaf bases retain humus for a long time. In the related genus *Drynaria* (Polypodiaceae; Figure 3X), humus-collecting leaves are completely differentiated from the green foliage leaves that produce spores—an example of holodimorphism (see section on sterile-fertile leaf dimorphy). The humus-collecting leaves are brown, stiff, papery, and dead at functional maturity. They grow over the fern's creeping rhizome, covering it completely. The leaf bases are sessile and wide so that the accumulated humus does not fall through. The humus-collecting leaves are much smaller than the green fertile ones, which also differ by being long-petiolate to elevate the sori away from the substrate where they are more likely to encounter air currents for spore dispersal. The related and widely cultivated staghorn ferns, *Platycerium* (Polypodiaceae), also have holodimorphic humus-collecting and fertile leaves. The humus-collecting leaves are also brown, stiff, papery, and dead at functional maturity. They grow tightly overlapped and appressed or ascending over rhizome, with sessile and broad bases—all modifications so that trapped organic matter does not fall through. The mesophyll is thick and, when dead at functional maturity, absorbs water like a sponge. In contrast, the fertile leaves are green, much longer, and arching away from the substrate. They are of relatively short duration on the plant, compared to the humus-collection leaves that persist for a long time. These drynarioid ferns provide an outstanding example of leaf modification as adaptations for life in the tree tops.

### Sterile-fertile leaf dimorphy

A special case of fern leaf morphological diversity is sterile-fertile leaf dimorphy. This phenomenon is generally thought to consist of narrower and taller fertile leaves compared to the sterile ones, but it is much more than that. Dimorphism is a syndrome of many characters, and these may be anatomical or morphological. The character differences maximize spore dispersal and minimize the metabolic cost of construction of fertile leaves (Moran, 1987). Narrower laminae or pinnae have a thinner boundary layer of air (Salisbury and Ross, 1992). This thinner layer of air would promote drying of sporangia and increase the chance that spores, after being catapulted from sporangia, would soon encounter moving air currents. Also maximizing spore dispersal are the longer and more erect petioles that elevate the fertile lamina away from the substrate where the spores are more likely to be picked up by air currents. After shedding their spores, fertile leaves have completed their function and soon wilt. Because fertile leaves are ephemeral relative to the sterile ones, they tend to be of “cheaper” construction, built with energetically less expensive tissues such as parenchyma and collenchyma, not harder and denser sclerenchyma.

Sterile-fertile leaf dimorphy is common in ferns. If sterile and fertile leaves are the same size and shape, they are said to be monomorphic. If differentiated, they are said to be dimorphic. The dimorphy may be of two types. In the first, only part of the leaf is fertile—the hemidimorphic condition—as exemplified by *Aglaomorpha meyeniana* (Polypodiaceae, Figure 3Y), *Belvisia* (Polypodiaceae, Figure 3M), *Osmunda* (Osumundaceae, Figure 3O), and Anemiaceae (Figure 3N). In the second, the complete fertile leaf is differentiated from the sterile—the holodimorphic condition—as seen in *Davallia heterophylla* (Davalliaceae, Figure 3K), *Matteuccia* (Onocleaceae, Figure 2M), and *Olfersia* (Dryopteridaceae, Figure 3L), *Osmundastrum* (Osmundaceae), and *Trachypteris* (Pteridaceae, Figure 3G). These two forms of dimorphy have evolved many times among ferns (Wagner and Wagner, 1977).

The fertile leaves of the sister families Psilotaceae and Ophioglossaceae are unusual in that the synangia in the former and the sporangia-bearing portion in the latter arise adaxially (Imaichi and Nishida, 1986; Wagner, 1990; Schneider, 2013). The fertile leaves of all other ferns bear sporangia abaxially (marginally in some groups such as the Hymenophyllaceae and Culcitaceae). Both families are further atypical in that a single vein runs to the base of the sporangium or synangium (Eames, 1936). In no other ferns are the sporangia similarly supplied.

Another aspect of dimorphy is the phenology of sterile and fertile leaf production (Sharpe and Mehltreter, 2010). This is especially unknown for tropical ferns not limited by an unfavorable winter growing season and thus capable of producing leaves throughout the year. In these cases, fertile leaves may be produced at certain times of the year, such as during the wet season or dry season. Also, in almost all tropical ferns with strong sterile-fertile leaf dimorphy, the fertile leaves tend to be shorter-lived than the sterile. Yet the timing of production and duration on the rhizome are unknown for all but a handful of ferns. Also, it is rarely known how many fertile leaves are produced annually in proportion to sterile ones. Phenology is one area of fern leaf biology where field studies are greatly needed.

### Foliar buds

About 5% of fern species worldwide have foliar-borne buds (Moran, 2004). Buds may form anywhere on the leaf: along the petiole, in the angle between the rachis and the pinnae, at the apices of the lamina or the pinnae, or above the point that sori would normally form. Buds contact the soil by various means. Most commonly, as the leaf senesces, the petiole weakens at the base, and the leaf gradually reclines toward the ground. Eventually the leaf comes to rest on the soil and the bud, still attached to the leaf, is “planted” and takes root. In other ferns, such as *Bolbitis heteroclita* (Dryopteridaceae, Figure 2O), *Thelypteris reptans* (Thelypteridaceae, Figure 2N) or *Asplenium rhizophyllum* (Aspleniaceae), the lamina apices are long-attenuate or flageliform (whip-like) with buds along their length or at their tips. These leaves arch over and touch the ground, placing the bud in contact with the soil. Rarely, foliar-borne buds abscise from the mature leaf and drop to the ground, as in *Cystopteris bulbifera* (Cystopteridaceae, Figure 3W).

### Rheophytic ferns

The leaves of rheophytic ferns exhibit several morphological and anatomical adaptations to their unusual habitat. Rheophytic species are those confined to the beds of swift-running streams and grow submerged during regularly occurring flash floods (Van Steenis, 1981; Kato and Iwatsuki, 1991). Some examples of rheophytic ferns are *Asplenium obtusifolium* (Aspleniaceae), *Osmunda lancea* (Osmundaceae), and *Tectaria lobbii* (Tectariaceae). These ferns are characterized by flood-resistant morphological features such as narrow leaf blades or narrow leaflets with cuneate bases (a phenomenon called stenophylly) and petioles that are flexible—both characters that reduce drag from rushing water. The root system is matted and tightly anchored to the substrate. Anatomically, fern rheophytes have a smaller mesophyll with more cells in the spongy layer and with fewer intercellular spaces compared to related upland species. The cuticle is thicker and the epicuticular wax deposits on the epidermis are denser. Finally, the frequency of occurrence of stomata per unit area of leaf is higher in rheophytes than in related upland species (Kato and Imaichi, 1992).

## Experimental analyses of fern leaf development

There is a rich history of experimental studies in ferns (reviewed in White, 1971; White and Turner, 1995). These studies were designed to understand how fern leaves, and ferns in general, develop. Experimental studies of fern leaves have investigated the growth of heteroblastic leaf sequences, cataphylls, and simple and compound leaves (Wardlaw, 1963; White, 1971). Here we will focus on leaf-development studies designed to understand phyllotaxy, adaxial/abaxial identity, and when and how a leaf is determined. These studies used microsurgery to isolate primordia from the shoot and/or adjacent leaves and also sterile techniques to grow leaves or shoot apices in isolation. Most studies use the terminology “P” and “I” where P1 is the youngest leaf primordium and P10 is the 10^th^ oldest primordium. The “I” indicates the incipient leaf primordia where I1 would be the first primordia to develop after P1, and I3 would be the third primordium to develop after P1. The positions of incipient primordia are estimated by the phyllotaxy of the species. Many of the experimental studies on fern leaf development that we will discuss were performed on two different leptosporangiate fern species *Dryopteris aristata* (Dryopteridaceae) and *Osmunda cinnamomea* (Osmundaceae). Both species have compound leaves. Although the former species is now classified in *Arachniodes* (Dryopteridaceae) and the latter in *Osmundastrum* (Osmundaceae), we will use here the older names employed by the researchers in the original developmental studies cited.

Fern leaves arise from the periphery of a shoot apical meristem (SAM) in a distinct phyllotaxy as do seed plant leaves (Wardlaw, 1963; Gifford and Foster, 1988). The SAM in ferns typically has 1 or 2 large apical cells surrounded by small cytoplasmically dense cells that divide frequently (Bower, 1884; Wardlaw, 1963; Bierhorst, 1971; White, 1971; McAlpin and White, 1974; Stevenson, 1976; White and Turner, 1995). Although the SAM of the ferns and seed plants differ anatomically (because of the presence of apical initial(s) in ferns), they are similar by having distinct zones of cells in the meristem (Wardlaw, 1963; White, 1971; McAlpin and White, 1974; Stevenson, 1976; Steeves and Sussex, 1989). Fern leaf primordia arise from one or a group of cells on the flank of the SAM (Bower, 1923; Wardlaw, 1949b; Steeves and Briggs, 1958; Bierhorst, 1977; Imaichi, 1980, 1982). A single apical cell forms at the tip of the leaf and has 2 cutting faces (Wardlaw, 1963; White and Turner, 1995). The leaf is formed by divisions of the apical initial as well as by divisions in the marginal meristem. Divisions in the marginal meristem and sub-marginal cells, and cessation of division in groups of cells regularly spaced along the marginal meristem, give rise to pinnae (Wardlaw, 1963). Similar to the fern SAM, the meristematic regions of the leaf also have distinct zones of cells, however, unlike the fern SAM the leaf meristems have a bilateral symmetry. Maturation of the developing fern leaf is acroscopic, that is, toward the apex (Figure 5). Its proximal portions mature first, with a wave of maturation proceeding distally (Briggs and Steeves, 1958; Voeller, 1960).

As in other vascular plants, the leaves of ferns are arranged in a fixed and predictive phyllotactic sequence around the shoot apex (Schoute, 1938; Gifford and Foster, 1988). Early experimental studies in leaf development were performed to understand how and what determined where a leaf developed on the flank of the SAM (White, 1971; Steeves and Sussex, 1989). In *Dryopteris aristata*, incisions were made to isolate incipient leaf primordia from the SAM and/or older primordia. The plants were allowed to grow and the resulting phyllotaxy was examined (Wardlaw, 1949a,b, c). This series of experiments showed that incisions around I1, disrupted the phyllotaxy, and the primordia that subsequently formed were misplaced. These studies supported earlier ones in angiosperms concluding that adjacent primordia influence the position of the incipient leaf primordia (Snow and Snow, 1932; Wardlaw, 1949a,b). The angiosperm data was interpreted as leaf primordia develop in the first available space on the meristem (Snow and Snow, 1932; Wardlaw, 1949a,b; Steeves and Sussex, 1989).

However, space is not limited in the *Dryopteris aristata* shoot tip compared to the angiosperm shoot tip, and therefore space constraints did not provide the best explanation for the control of phyllotaxy in ferns (Wardlaw, 1949a,b; Steeves and Sussex, 1989). In addition, in the *Dryopteris aristata* phyllotaxy experiments, the “misplaced” primordia grew faster than the adjacent older leaves, which suggested that nearby older leaves inhibit the growth of younger leaves (Wardlaw, 1949a,b,c). Therefore, the phyllotaxy experiments in ferns were better interpreted using the field theory of phyllotaxy as opposed to being directed by available space (Wardlaw, 1949a; White, 1971; Steeves and Sussex, 1989). This theory states that there are regions of inhibition around the SAM and around the leaves and those regions are the ones that control when and where a leaf primordium develops (Wardlaw, 1949a,b,d; White, 1971; Steeves and Sussex, 1989). Experimental studies have shown that older leaves not only inhibit the growth and placement of nearby incipient leaf primordia but also have a role in determining whether a leaf primordium develops as a leaf or a shoot (reviewed in White, 1971).

Experiments were performed to understand the relationship between the SAM and leaves in ferns. In *Dryopteris aristata, *if all of the surrounding primordia are removed from a growing shoot tip, then the apical meristem continues to grow and produce leaves (Wardlaw, 1947, 1949a,c; White, 1971). In addition, isolated SAMs grown in sterile culture on media supplemented with sucrose and auxin formed adult plants (White, 1971). The results of these experiments indicate that the SAM is capable of autonomous development and there are no signals coming from leaves that direct shoot development. Experiments were also developed to understand how a determinate dorsiventral leaf arises from an indeterminate radial SAM. Sterile culture and microsurgery experiments were designed to understand whether the signals for a leaf to develop as a leaf come from the SAM, the developing leaf itself, or the surrounding leaves.

Microsurgery experiments have provided further insight into the relationship between the shoot apical meristem and leaf development. If a shallow incision is made between an incipient leaf primordium and the apex of the SAM, then the primordium still develops as a leaf (Wardlaw, 1956). However, if a deep incision separates an incipient leaf primordium and the SAM, then the incipient leaf primordium develops as a shoot (Wardlaw, 1949b). These results suggest that a leaf determining signal comes from the SAM and that fern leaves are not determined as leaves immediately upon arising from the SAM. To better understand when a leaf is determined as a leaf, deep incisions were made between leaf primordia in *Dryopteris aristata* at various developmental stages (Cutter, 1954, 1956; White, 1971). If incisions were made between the SAM and the leaf primordia at developmental stage P1, then these isolated primordia were more likely to grow out as buds (Cutter, 1954, 1956). However, if incisions were made between the SAM and leaf primordia at developmental stage P4, then these isolated primordia were more likely to grow out as leaves (Cutter, 1954, 1956). Leaf primordia P1, P2, and P3 are more plastic in their development and after incisions, may grow out as buds, although a few still develop as leaves. These experiments indicate that a *Dryopteris aristata* leaf is determined as a leaf sometime after stage P1. Although some results showed that if I1 is isolated then it more likely develops as a bud, but in rare cases it developed as a leaf (Amer and Williams, 1957). Similar results were found in *Osmunda cinnamomea* leaf primordia at developmental stages P1-P10 that were isolated and grown in sterile culture (Steeves, 1961, 1963; White, 1971). These excised leaves varied in their development, but generally the isolated younger primordia developed as shoots and the older ones as leaves.

Sterile culture experiments were also used to investigate if a leaf determining signal also came from leaves. In these experiments, *Osmunda cinnamomea* leaf primordia were excised and grown with other leaves. If isolated P3 leaves, which are still plastic in their developmental potential, were grown with older leaves (isolated P10 or P12 leaves), then the P3 leaves were more likely to grow out as leaves instead of buds. (Kuehnert, 1967, 1969a,b,c; Haight and Kuehnert, 1969; White, 1971). However, if isolated P3 leaves are grown with mature P14 leaves, then the P3 leaves mostly developed as buds (Kuehnert, 1969a,b; White, 1971). These experiments indicate that a leaf determining signal is coming from older yet still developing leaves. Further sterile culture experiments were performed to determine if the signal coming from leaves involved a diffusible substance. Isolated P3 primordia were grown on media containing homogenized leaves (P10 or P12), which resulted in most of P3 primordia developing as leaves (Kuehnert, 1969b; White, 1971). These results are similar to those found with isolated P3 primordia grown with P10 or P12 leaves. Isolated P3 leaves were also grown with older leaves that were not in physical contact with each other. In these experiments the leaf pairs were separated by either an impermeable barrier or a permeable barrier. The results of these experiments were similar to the previous experiments that show that the older primordia have a leaf determination effect on P3 and that the determination effect appears to be mediated by a diffusible substance.

In the experiments with *Osmunda cinnamomea*, most of the excised P3 leaves develop as buds; however, some still develop as leaves. This suggests that another part of the plant has a determining influence on leaf development besides older leaves. If all of the leaf primordia are removed from the SAM, then the incipient leaf primordium (I1) develops as a leaf (Hicks and Steeves, 1969; White, 1971). In addition, if I1 is isolated from the apex then the incipient primordium develops as a shoot. These experiments indicate that the SAM is not only capable of autonomous development but also has a leaf-determining influence on incipient primordia.

A defining characteristic of nearly all vascular plant leaves is that they have adaxial/abaxial identities. This distinguishes leaves from shoots, which are radially symmetric. However, Wardlaw (1949b,d) concluded that initially leaf primordia and shoot buds of *Dryopteris aristata* are histologically indistinguishable. In some fern microsurgery experiments where leaf primordia (P4-P9) were isolated from the SAM by incisions, some determinate leaves grew out that had near radial symmetry and buds in their axils (Wardlaw, 1945, 1947, 1949c; Cutter, 1954). Similar results were found in angiosperms where incisions separating I1 from the shoot apex resulted in the development of radially symmetric organs (Snow and Snow, 1935; Sussex, 1951). The depth of the incisions and the location of the cuts had an impact on the fate of I1 (Sussex, 1951; Cutter, 1954). If incisions were made close to I1 then the primordia that grew out were radially symmetric. These microsurgery experiments in ferns and angiosperms indicated that the shoot apical meristem had an influence on the adaxial/abaxial identity of leaves (Sussex, 1951; Cutter, 1954).

In summary, the microsurgery experiments in ferns supported the field theory of phyllotaxy; that is, there is a field of inhibition around the SAM and existing primordia that prevents incipient leaf primordia developing too closely to these fields. Microsurgery and sterile culture experiments demonstrated that the SAM does not require attached leaf primorida or leaves to continue the development of the adult shoot and therefore the SAM is capable of autonomous development. Microsurgery and sterile culture experiments also revealed that the SAM and developing leaves influence the developmental fate of leaf primordia. In addition, the signal from developing leaves involves a diffusible substance. Finally, the SAM was found to play a role in the specification of adaxial/abaxial identity in leaves. Molecular genetic studies in angiosperm leaf development are beginning to provide clues into the nature of the signaling that occurs between the shoot apex and leaf primordia (Byrne, 2012). Auxin signaling has been shown to play an integral role in angiosperm leaf development (Byrne, 2012; Traas, 2013). In angiosperms, polar auxin transport is important in specifying phyllotaxy, leaf patterning, and is integral in the leaf development network involving 2 key transcription factors, Class I KNOX and Class III HD-Zip (see below). The experimental evidence indicates that similar signaling occurs between the shoot apex and leaf primordia in ferns, however, little is known about the molecular nature of these signals in ferns. Auxin is a likely candidate for signaling in fern leaf development. Auxin has been shown to effect the leaf complexity in the fern *Marsilea* as well as crozier development (Allsopp, 1952; Steeves and Briggs, 1960). However, much work remains to be done on the molecular genetics of leaf development in ferns.

## Molecular genetics of fern leaf development

Much is known about the molecular genetics of the leaf developmental network in angiosperms (Byrne, 2012). However, outside of the seed plants, comparative molecular studies so far have focused on only two transcription factors: Class I KNOX and Class III HD-Zip (Harrison et al., 2005; Prigge and Clark, 2006; Floyd and Bowman, 2006). In all vascular plants, leaves arise from the SAM. Two criteria can be used to differentiate the leaf from the shoot in most vascular plants: determinacy and adaxial/abaxial polarity (Arber, 1941). Interestingly, these are exactly the roles played by Class I KNOX and Class III HD-Zip genes, respectively. These two genes have been used in comparative studies to better understand leaf evolution and development in vascular plants. Class I KNOX genes, have important roles in maintaining the indeterminacy of the SAM (reviewed in Hay and Tsiantis, 2010; Byrne, 2012; Townsley and Sinha, 2012). They are downregulated in determinate simple leaves but upregulated in compound leaves to specify pinnae (Bharathan et al., 2002; reviewed in Hay and Tsiantis, 2009). Class III HD-Zip proteins have important roles in the development of the SAM, vasculature, and the adaxial region of the leaf in flowering plants (Prigge et al., 2005; Floyd and Bowman, 2006, 2007). The specification of adaxial and abaxial identities has been suggested to be important for lamina outgrowth (Waites and Hudson, 1995). Therefore, we know the genes that are important for specifying determinacy and polarity—two key characteristics of leaves, and can study the role of these two genes in vascular plants to better understand the evolution and development of leaves.

Class I KNOX and Class III HD Zips genes have been well-studied across the flowering plants and to a lesser extent in gymnosperms (reviewed in Floyd and Bowman, 2007; Efroni et al., 2010; Floyd and Bowman, 2010; Hay and Tsiantis, 2010; Byrne, 2012; Townsley and Sinha, 2012; Yamaguchi et al., 2012). Studies of Class I KNOX and Class III HD Zip expression have also been performed in lycophytes. These studies came to diametrically opposite conclusions about the conservation of a leaf developmental mechanism between microphylls and megaphylls (Harrison et al., 2005; Floyd and Bowman, 2006; Prigge and Clark, 2006). The expression of Class III HD Zip genes have not been studied in ferns; however, there have been several studies of Class I KNOX gene expression in ferns. Because ferns occupy a key phylogenetic position as sister to the seed plants (Figure 1), comparative studies in diverse fern species may help to elucidate the evolution of these leaf developmental regulators and their role in leaf development.

Comparative studies of the Class I KNOX genes have been performed in the leptosporangiate ferns *Anogramma chaerophylla* (Pteridaceae), *Ceratopteris richardii* (Pteridaceae), and *Osmunda regalis* (Osmundaceae, Figure 3O), all of which have divided leaves (Bharathan et al., 2002; Harrison et al., 2005; Sano et al., 2005). These studies found that, as in seed plants with compound leaves, Class I KNOX genes are expressed in the meristem and in the developing leaves. Yet unlike seed plants, Class I KNOX genes were not found to be down-regulated in leaf primordia. These results might suggest either the independent origin of megaphylls in ferns and seed plants, or may simply reflect the prolonged indeterminacy of fern leaves. In flowering plants, Class I KNOX genes are down-regulated while ARP genes are up-regulated in leaf primordia (reviewed in Floyd and Bowman, 2007, 2010; Hay and Tsiantis, 2010; Efroni et al., 2010). In addition to Class I KNOX expression, ARP protein expression was also studied in *Osmunda* (Harrison et al., 2005). This study found that the expression of Class I KNOX and ARP genes overlapped in the meristem and leaves unlike the complementary expression patterns in flowering plants (Harrison et al., 2005). In support of their results in lycophytes, Harrison et al. (2005) suggested that this may reflect the ancestral role of the leaf developmental module of Class I KNOX and ARP in shoot branching, and that this module was recruited independently during leaf evolution in vascular plants.

## Future directions in fern leaf evo-devo

The results of molecular genetic studies of KNOX/ARP in *Osmunda* and the KNOX regulatory network in compound-leaved angiosperms have brought the partial shoot theory of Agnes Arber back into discussions of leaf evolution and development (Arber, 1941; Barkoulas et al., 2007; Koenig et al., 2009). Arber considered the shoot to be the fundamental organ of the plant, and that all leaves are “partial shoots, and only partial shoots” because their indeterminate growth and radial symmetry are repressed (Arber, 1941).

The morphology of fern leaves, in particular, has shoot-like characteristics. Fern leaves have extended indeterminacy, and some have indeterminate leaves (see Leaf indeterminacy section above). Anatomically, Wardlaw (1949b,d) found the shoot and leaf primordia indistinguishable from each other. In addition, experimental studies found that fern leaves are not determined as leaves until later in their development but when first specified have more of a shoot identity (reviewed in White, 1971). Further support for the shoot-like characteristics of fern leaves comes from recent studies in *Nephrolepis exaltata* (Nephrolepidaceae) that found that there is a reiteration from shoot apical meristem to leaf to pinnae that suggests a reiteration of a shoot program (Sanders et al., 2011).

The results from experimental leaf studies performed in ferns parallel the results found in angiosperms. These results suggest that similar developmental processes are at work in both plant groups, furthermore paleobotany studies suggest that these processes may not be occurring in the same sequence or have similar timing (Boyce and Knoll, 2002; Sanders et al., 2009). Molecular genetic studies of leaf evo-devo in ferns could not only fill the gap that exists in our understanding of fern leaf development, but also provide crucial data to the debate on the evolution and origin of megaphylls. For example, developmental genetic studies in ferns with diverse morphologies (e.g., simple vs. compound leaves) could provide the molecular basis for their morphological diversity. In addition, studies of leaf development genes in *Psilotum* (Psilotaceae), *Ophioglossum* (Ophioglossaceae), and *Equisetum* (Equisetaceae) could provide crucial molecular data to better interpret the leaf morphology of these distinctive genera. A vast amount of fern research has been performed in the fields of paleobotany, morphology, development, and experimental biology. The field of developmental genetics is the missing piece and when integrated with data from other fields will help us develop more robust hypotheses of fern leaf evolution and development and more broadly hypotheses of leaf evolution and development in vascular plants.

### Conflict of interest statement

The authors declare that the research was conducted in the absence of any commercial or financial relationships that could be construed as a potential conflict of interest.
